# A Case of Binge Drinking with Comorbid Anxiety Disorder, Premenstrual Dysphoric Disorder, and Emotional Eating Treated with Fluoxetine and Acamprosate

**DOI:** 10.1192/j.eurpsy.2026.10839

**Published:** 2026-07-31

**Authors:** S. Özcelep, M. H. Aksu

**Affiliations:** Psychiatry, Gazi University Hospital, Ankara, Türkiye

## Abstract

**Introduction:**

Episodic binge drinking, characterized by periods of heavy alcohol use separated by abstinent intervals, is not explicitly defined in DSM-5 but may represent a pattern within *Alcohol Use Disorder (AUD).* Diagnostic complexity increases when comorbid conditions such as generalized anxiety disorder, premenstrual dysphoric disorder (PMDD), and disordered eating behaviors are present.

**Objectives:**

To present a case of a young woman with episodic binge drinking, comorbid anxiety disorder, PMDD, and emotional/night eating. The aim was to highlight anxiety as a trigger for binge episodes and describe the benefit of fluoxetine and acamprosate.

**Methods:**

A descriptive case report was prepared, including psychiatric, family, and developmental history, clinical features, and treatment course. DSM-5 criteria were applied to evaluate comorbid conditions.

**Results:**

The patient is a 29-year-old single female designer with a nine-year history of episodic binge drinking. Months of abstinence were interrupted by 1–2 weeks of uncontrolled alcohol use (>10 beers/day, sometimes spirits), causing blackouts, conflict, and withdrawal (tremor, palpitations, perceptual disturbances). Episodes were consistently preceded by anxiety symptoms such as restlessness, fear of nightmares, insomnia, and autonomic arousal, suggesting alcohol functioned as a maladaptive coping strategy. Attacks were also temporally associated with PMDD. Additional features included night eating and emotional eating, overlapping with DSM-5 *Binge-Eating Disorder* and *Other Specified Feeding or Eating Disorder (OSFED).* She reported weight concerns. Psychiatric history revealed generalized anxiety disorder with partial response to sertraline. Anamnesis showed a tense relationship with her father, who appeared cheerful during intermittent alcohol use, shaping early associations between alcohol and positive affect. Family history was also notable for maternal anxiety disorder. Alcohol misuse negatively affected romantic relationships, without legal or financial issues. Treatment with fluoxetine 40 mg/day and acamprosate (666 mg tablet, 2 tablets three times daily; total 1998 mg/day) led to marked reduction in anxiety, alcohol craving, emotional eating, and insomnia. At 12-week follow-up, interpersonal functioning improved and no new binge episodes occurred.

**Conclusions:**

This case shows the importance of recognizing episodic binge drinking as a clinically relevant pattern of AUD and its interplay with comorbid anxiety, PMDD, and disordered eating. The consistent presence of anxiety before binge episodes suggests alcohol was used as self-medication. Response to fluoxetine and acamprosate highlights the value of integrated treatment addressing both anxiety and craving. Systematic reporting may help clarify the classification of episodic binge drinking and guide management strategies.

**Disclosure of Interest:**

None Declared

